# Mechanical Circulatory Support for High-Risk Percutaneous Coronary Intervention: Is It a Choice or Chance That Determines Outcomes?

**DOI:** 10.1016/j.jscai.2025.104021

**Published:** 2025-11-04

**Authors:** Ali O. Malik, Jason R. Wollmuth

**Affiliations:** a Department of Interventional Cardiology, Fairfield Medical Center, Lancaster, Ohio; b Department of Interventional Cardiology, Providence Heart Institute, Portland, Oregon

**Keywords:** high-risk, mechanical circulatory support, percutaneous coronary intervention

Percutaneous coronary intervention (PCI) in high-risk patients based on anatomy (left main disease, multivessel disease, calcification), comorbidities (advanced age, frailty, renal dysfunction, valvular heart disease), and adverse hemodynamics (decreased left ventricle ejection fraction, poor cardiac output, relative hypotension)^1^ poses a significant challenge for interventional cardiologists. Although generally well tolerated, alterations in coronary perfusion during PCI in high-risk patients can limit quality and completeness of revascularization, potentially leading to worse short-term and long-term outcomes. Use of mechanical circulatory support (MCS) devices can minimize hemodynamic compromise and enable optimal completion of PCI.^2^

In contemporary practice, there are 2 MCS devices that are primarily used and widely available: percutaneous ventricular assist device (PVAD) and intra-aortic balloon pump (IABP). The use of IABP in high-risk PCI has not been shown to improve patient outcomes,^3^ and a randomized trial comparing PVAD and IABP for high-risk PCI did not show any differences in short-term outcomes.^4^ This comparison, however, was not sufficiently powered to examine intermediate-term outcomes and did not account for the learning curve associated with PVAD use,^5^ and a per-protocol analysis did show a trend toward reduction in major adverse events at 90 days with the use of PVAD.^6^ Despite limited randomized trial data, IABP is still commonly used, and there has been an increase in the use of PVAD for PCI in high-risk patients in recent years.^3^

In this issue of *JSCAI*, Shah et al^7^^,^^8^ present 2 articles examining the use of PVAD and IABP for high-risk PCI.^7^^,^^8^ In the research letter,^8^ the authors highlight limitations in comparing outcomes among patients receiving PVAD- and IABP-supported PCI using observational data. The authors highlight that in real-world use, there is no equipoise in device selection for high-risk PCI. Rather, there appears to be preferential use of PVAD for very high-risk patients and IABP for patients at a relatively lower risk. This is illustrated in the propensity score matching analysis, showing minimal overlap at the extremes of risk. As the authors point out, any observational analysis comparing outcomes between the use of PVAD and IABP electively for PCI will likely exclude or heavily discount patients at the extremes of risk. The authors also describe other challenges with observational comparison from administrative databases, such as inability to distinguish between prophylactic use and bailout; bias due to confounders, risk modifiers, and colliders; and inability to completely adjust for the inherent selection bias.

Keeping in mind the abovementioned limitations, the authors’ leveraged data from a contemporary administrative database (2018-2024) to compare outcomes with the use of PVAD and IABP for high-risk patients (without shock or ST-segment myocardial infarction) undergoing elective PCI.^6^ The strength of this analysis is a large, contemporary (2018-2024), real-world database of more than 1000 hospitals and hospital systems, not only covering approximately 25% of all inpatient hospital admissions throughout of the United States but also using a sound statistical framework and conclusions. The primary limitation of this work is inherent to all observational studies—unaccounted selection bias despite statistical adjustments used for the analysis. Moreover, with the strict inclusion and exclusion criteria, there were a significant number of patients who were excluded from the analysis. Of 148,791 patients in the database, only 4781 met the selection criteria (3859 PVAD and 922 IABP). After propensity score matching analysis, 847 IABP-treated patients were matched with 2416 PVAD-treated patients. PVAD-treated patients had a lower incidence of the primary end point (the 90-day mortality) than IABP-treated patients (7.9% vs 11.8%; *P* = .011) and had a lower rate of 90-day major adverse cardiac and cerebrovascular events (composite of mortality, stroke, and myocardial infarction at 90 days; 10.3% vs 14.9%; *P* < .001). These observations are important and inform the readers about real-world outcomes of patients without shock who undergo MCS-assisted PCI with contemporary devices.

In conclusion, we commend the authors for their rigorous analysis while highlighting the inherent limitations in the dataset. Their analysis serves as a blueprint for future observational comparisons for the use of MCS devices for high-risk PCI. While the present analysis shows improved outcomes in high-risk patients undergoing PCI with PVAD compared with those in patients undergoing IABP, until there is a randomized controlled trial, any final conclusions cannot be reached. The authors’ analysis highlighted the preferential use of PVAD over IABP in higher-risk patients. This perceived lack of equipoise is important to note as this may impact clinicians’ willingness to enroll patients in future clinical trials of MCS in high-risk PCI. What determines relevant patient outcomes? Is it choice of MCS device or chance? As we await the results of the upcoming randomized controlled trials, the jury remains out.

## References

[1] Riley R.F., Henry T.D., Mahmud E. (2020). SCAI position statement on optimal percutaneous coronary interventional therapy for complex coronary artery disease. Catheter Cardiovasc Interv.

[2] Zeitouni M., Marquis-Gravel G., Smilowitz N.R. (2022). Prophylactic mechanical circulatory support use in elective percutaneous coronary intervention for patients with stable coronary artery disease. Circ Cardiovasc Interv.

[3] Perera D., Stables R., Thomas M. (2010). Elective intra-aortic balloon counterpulsation during high-risk percutaneous coronary intervention: a randomized controlled trial. JAMA.

[4] O’Neill W.W., Kleiman N.S., Moses J. (2012). A prospective, randomized clinical trial of hemodynamic support with Impella 2.5 versus intra-aortic balloon pump in patients undergoing high-risk percutaneous coronary intervention: the PROTECT II study. Circulation.

[5] Henriques J.P., Ouweneel D.M., Naidu S.S. (2014). Evaluating the learning curve in the prospective randomized clinical trial of hemodynamic support with Impella 2.5 versus intra-aortic balloon pump in patients undergoing high-risk percutaneous coronary intervention: a prespecified subanalysis of the PROTECT II study. Am Heart J.

[6] Dangas G.D., Kini A.S., Sharma S.K. (2014). Impact of hemodynamic support with Impella 2.5 versus intra-aortic balloon pump on prognostically important clinical outcomes in patients undergoing high-risk percutaneous coronary intervention (from the PROTECT II randomized trial). Am J Cardiol.

[7] Shah T., Holy C., Almedhychy A. (2025). Safety and efficacy of percutaneous ventricular assist device versus intra-aortic balloon pump in high-risk PCI. J Soc Cardiovasc Angiogr Interv.

[8] Shah T., Holy C., Almedhychy A. (2025). Pitfalls in comparing mechanical circulatory support devices using administrative datasets: epidemiology of the diseased population. J Soc Cardiovasc Angiogr Interv.

